# Extended Field of View and Resolution Enhancement in Lensless Digital Holography

**DOI:** 10.3390/s26092821

**Published:** 2026-04-30

**Authors:** Chung-Hsuan Huang, Chih-Cheng Hsu, Huai-Che Chu, Chau-Jern Cheng, Han-Yen Tu

**Affiliations:** 1 Department of Electrical Engineering, Chinese Culture University, Taipei 11114, Taiwan; hcx4@ulive.pccu.edu.tw (C.-H.H.); b2208526@ulive.pccu.edu.tw (C.-C.H.); 2 Institute of Electro-Optical Engineering, National Taiwan Normal University, Taipei 11677, Taiwan; 61277018h@ntnu.edu.tw (H.-C.C.); cjcheng@ntnu.edu.tw (C.-J.C.)

**Keywords:** lensless digital holography, extended field of view, resolution enhancement

## Abstract

Lensless digital holography provides a simple, low-cost imaging platform with a large field of view (FOV) and quantitative phase capability, making it attractive for biomedical imaging, microstructure inspection, and large area imaging. However, the achievable FOV is still limited by sensor size, and in-line reconstruction suffers from twin-image artifacts that degrade image quality. To overcome these limitations, this study proposes an extended FOV lensless digital holography method that combines hologram stitching with multi-depth phase retrieval. Multiple holograms acquired from laterally shifted FOVs are stitched to form an extended hologram, while holograms recorded at multiple axial depths are used to suppress twin-image artifacts and improve reconstruction fidelity. Experimental results show that the proposed method effectively expands the imaging area, enhances effective resolution by integrating complementary diffraction information from different FOVs, and improves image contrast and feature visibility. This approach enables extended FOV, resolution enhancement, and high-quality holographic imaging while preserving the simple lensless digital holography architecture.

## 1. Introduction

Lensless digital holography [1,2,3,4] has emerged as a representative computational imaging technique in recent years, owing to its ability to reconstruct both amplitude and phase information without the use of conventional imaging lenses. In lensless digital holography, in-line holograms are directly recorded by an image sensor, and the wavefront of the object is numerically reconstructed through wave propagation and reconstruction algorithms. Compared with conventional digital holographic microscopy systems that rely on high-magnification objective lenses, lensless digital holography greatly simplifies the optical configuration while offering several significant advantages, including a large field of view (FOV), compact system design, high mechanical stability, and low cost. These characteristics make lensless digital holography particularly suitable for large area imaging applications, where conventional optical systems are often limited by field coverage, system complexity, or acquisition efficiency.

Among its diverse applications, biomedical imaging [5,6] and point-of-care diagnostics [7,8] represent some of the most prominent directions for lensless digital holography. Because its imaging area can typically approach the effective area of the image sensor, lensless digital holography enables the simultaneous observation of a large number of biological specimens in a single exposure, including red blood cells, white blood cells, bacteria, parasites, and cultured cells [9,10]. When combined with phase retrieval techniques, such as multi-wavelength [11,12] or multi-depth [13,14] iterative algorithms, single-shot propagation-constrained phase retrieval, and deep learning-based approaches, lensless digital holography can provide not only quantitative phase distributions of cells but also estimates of morphological features, dry mass, and other physical parameters related to refractive index distributions [15,16,17], thereby enabling label-free cellular analysis. These characteristics make lensless digital holography particularly attractive for long-term live cell monitoring, drug response analysis, and clinical testing or on-site diagnostics in resource-limited settings.

Despite its large FOV capability, the spatial resolution of lensless digital holography remains constrained by several factors, including sensor pixel size, illumination wavelength, propagation distance between the object and the sensor, and the performance of the reconstruction algorithm [18]. In addition, because in-line holographic recording is inherently susceptible to twin-image interference, the reconstructed image quality is often degraded by background artifacts, reduced contrast, and limited visibility of fine structures. To overcome these limitations in resolution, reconstruction quality, and imaging performance, various enhancement strategies have been proposed in recent years. For example, pixel super-resolution methods [19,20] improve the sampling limitation of the sensor by integrating multiple low resolution holograms with subpixel shifts. Synthetic aperture methods [21,22] combine holograms recorded under different illumination angles or detector positions to extend the effective spatial frequency bandwidth and thereby improve imaging resolution. Furthermore, deep learning-based phase retrieval and image reconstruction methods [23,24] can generate high-quality amplitude and phase images with high computational efficiency under specific training conditions. Although these approaches have shown considerable potential in improving resolution, suppressing artifacts, or accelerating reconstruction, they generally require additional hardware control, illumination modulation, or large amounts of training data, and most of them primarily focus on resolution enhancement or reconstruction quality improvement within a single FOV.

Although lensless digital holography inherently provides a large FOV, the imaging area is still ultimately limited by the finite sensor size. When the sample extends beyond the coverage of a single sensor, image stitching techniques are typically required to achieve large area imaging. To address this limitation, various FOV extension strategies have been proposed in digital holography, particularly through multiplexing-based approaches and multi-channel imaging architectures. For example, off-axis holographic multiplexing has been used to encode multiple complex wavefronts with different fringe orientations in a single camera exposure, enabling rapid wavefront acquisition and processing [25]. Spatial multiplexing methods have also been demonstrated to double the FOV of off-axis digital holography by encoding two different object regions into a single hologram [26,27]. More recently, angular- or polarization-based multiplexing configurations have further extended the FOV or enabled double-FOV polarization-sensitive holographic imaging [28,29]. These studies have demonstrated that such approaches can effectively increase imaging coverage while maintaining imaging performance. However, these multiplexing-based strategies typically require additional optical components, carefully designed illumination configurations, or system-level modifications to enable multiplexing and signal separation, which increases system complexity and may limit their flexibility in practical implementations. Alternatively, image stitching provides a purely computational approach for extending the FOV. Existing image stitching methods include feature-based stitching [30], phase correlation [31], mutual information [32], complex-field stitching [33], and deep learning-based methods [34], which have demonstrated improved robustness under challenging imaging conditions. However, most existing studies primarily focus on stitching in-focus images or reconstructed wavefront maps, where structural features are more distinguishable and local contrast is sufficient for reliable matching. In contrast, stitching defocused holographic diffraction patterns in lensless digital holography remains a challenging and relatively underexplored problem. Unlike in-focus images, defocused holograms exhibit low contrast and highly repetitive fringe patterns, making feature extraction and reliable registration significantly more difficult. More importantly, conventional stitching strategies are mainly designed to extend spatial coverage, rather than to enhance the intrinsic resolution of the imaging system. From a computational imaging perspective, however, stitching multiple defocused holograms acquired from different lateral positions provides an opportunity to integrate complementary spatial frequency components, enabling large FOV hologram synthesis and potentially improving the effective reconstruction resolution beyond that achievable from a single view.

Nevertheless, stitching defocused holographic diffraction images in lensless digital holography is highly challenging because the recorded holographic patterns are often similar and exhibit relatively low texture. Dense Kernelized Feature Matching (DKM) [35], recently proposed as a dense image matching framework, offers a promising solution to this problem. Unlike conventional approaches that rely on a limited number of sparse keypoints, DKM directly estimates dense pixel-wise correspondences between two images. Through a coarse-to-fine matching strategy, it first establishes global correspondences, then performs local refinement, and simultaneously estimates matching confidence. As a result, DKM has demonstrated strong robustness in challenging image scenarios with low texture, repetitive patterns, or weak local features. These properties make DKM particularly suitable for aligning defocused holographic diffraction patterns across multiple views, thereby facilitating reliable multiple holograms stitching for large area reconstruction.

In this study, a unified framework is proposed for extended FOV and resolution enhancement lensless digital holography imaging by integrating multiple holograms stitching with multi-depth phase retrieval. First, defocused holographic diffraction images recorded from different FOVs are stitched using the DKM model to extend the imaging area and integrate complementary diffraction information from multiple FOVs. Subsequently, holograms at multiple axial positions are acquired through controlled sensor displacement, and the same stitching strategy is applied to construct multi-depth hologram datasets. Finally, a multi-depth phase retrieval method is employed to iteratively reconstruct the complex wavefront, enabling improved phase accuracy and effective suppression of twin-image artifacts. The proposed multi-depth phase retrieval for the extended field of view (MD-EFOV) framework in lensless digital holography achieves synergistic enhancement in imaging coverage, reconstruction fidelity, and effective resolution, offering a practical and scalable solution for large area quantitative phase imaging.

## 2. Working Principle

### 2.1. Flowchart of MD-EFOV Lensless Digital Holography

**Figure 1:**
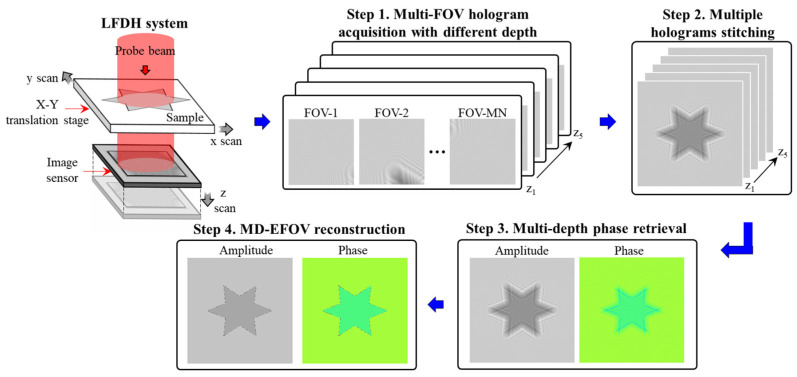
Flowchart of lensless digital holography imaging with extended field of view and resolution enhancement.

To achieve extended FOV and resolution enhancement in lensless digital holography, this study integrates multiple holograms stitching with multi-depth phase retrieval. The overall workflow is shown in Figure 1 and consists of four main steps, as detailed below.

- Step 1: Multi-FOV hologram acquisition with different depth

To capture object information over an extended FOV, multiple holograms are recorded by laterally scanning the sample using a motorized translation stage. During acquisition, the sample is sequentially shifted across different positions in the x-y plane, resulting in a set of partially overlapping fields of view. This strategy enables large area imaging while preserving the simplicity of the lensless digital holography system, and allows the integration of complementary diffraction information across different FOV. After completing the x-y scanning and holograms acquisition at a given axial position, the image sensor is translated along the *z*-axis using a motorized stage to a new depth plane. The x-y scanning process is then repeated to acquire another set of holograms. By iteratively adjusting the sensor position along the optical axis, multiple holograms datasets at different depths are obtained. In this study, a total of five axial planes are recorded to construct a multi-depth holographic dataset, which is essential for robust phase retrieval and improved reconstruction fidelity.

- Step 2: Multiple holograms stitching

To accurately estimate the spatial relationships among different FOVs acquired during scanning, the DKM method is employed for multiple holograms stitching. To avoid intensity discontinuities caused by nonuniform optical field distribution, image blending is applied to the overlapping regions between adjacent FOVs during stitching. This process produces an extended FOV defocused holographic diffraction image. The proposed stitching strategy not only suppresses phase discontinuities and brightness mismatch at the stitching boundaries but also improves the overall stability of the stitched result and the subsequent reconstruction quality. The details of the stitching strategy and framework are described in Section 2.2 and Section 2.3.

- Step 3: Multi-depth phase retrieval

To suppress the twin-image artifacts commonly encountered in in-line holography and to improve reconstruction accuracy, a multi-depth phase retrieval method is adopted. By incorporating holograms recorded at multiple axial planes, the proposed method performs iterative wavefront propagation with amplitude constraints across different depths to recover a more accurate and stable complex wavefront distribution. The integration of multi-depth information provides additional physical constraints that effectively mitigate twin-image interference and reduce reconstruction ambiguity. As a result, both amplitude and phase reconstructions are significantly improved in terms of accuracy and robustness. Detailed implementation of the multi-depth phase retrieval method is presented in Section 2.4.

- Step 4: MD-EFOV reconstruction

In the final stage, the stitched and phase-retrieved MD-EFOV complex wavefront is numerically propagated to the target reconstruction plane using the angular spectrum method. This step enables the recovery of the object’s complex wavefront distribution at the in-focus plane. Through the proposed MD-EFOV reconstruction framework, the system simultaneously achieves large area imaging, resolution enhancement, and high-quality quantitative phase reconstruction. This unified approach provides a practical solution for lensless digital holography applications requiring both wide coverage and high reconstruction fidelity.

### 2.2. Multiple Holograms Stitching

**Figure 2:**
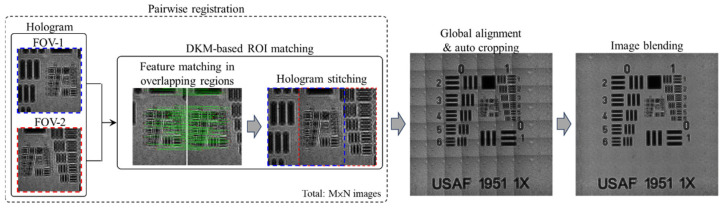
Workflow of multiple holograms stitching.

The workflow of the proposed multiple holograms stitching is shown in Figure 2. Its purpose is to achieve robust registration and stitching of holograms acquired from adjacent FOVs, thereby constructing an extended FOV hologram. The overall procedure consists of four main steps, including x-y scanning coordinate establishment, pairwise registration, global alignment and auto cropping, and image blending, as described below.

First, in the x-y scanning stage, the system reads each hologram file and extracts the recorded x-y physical coordinates to establish the relative positions of the holograms and the grid topology over the scanning plane. Through this step, the adjacency relationships among different FOVs can be explicitly determined, providing the basis for subsequent adjacent-image pairing and stitching order arrangement.

Next, in the pairwise registration stage, local feature matching and relative shift estimation are performed for each pair of adjacent holograms based on their overlapping regions. Specifically, according to the physical overlap ratio defined by the actual scanning process, the overlapping regions between adjacent holograms are extracted. These regions are then used as the regions of interest (ROIs) for model input. To account for stage positioning errors and potential loss of boundary features, an additional search margin of approximately ±10% is reserved around the theoretical overlap region, thereby improving the tolerance and robustness of the matching process. In this study, feature matching and local stitching within the overlapping regions are performed using the DKM method to improve registration stability under low contrast conditions, repetitive textures, and complex diffraction fringes. Compared with directly matching the entire hologram, this strategy effectively reduces computational cost while improving the reliability of local alignment.

After feature matching is completed in the overlapping regions, the system further calculates the relative shift vector (Δx, Δy) for each adjacent image pair, including both horizontal and vertical neighboring pairs. Because the matching results may contain a certain number of mismatched correspondences, all estimated shifts must be statistically analyzed and filtered to obtain a stable pairwise shift. First, the median of all matched displacements is used as an initial estimate to reduce the influence of outliers. Then, all displacement vectors are projected onto a two-dimensional histogram, and the dominant displacement direction with the highest number of votes is identified as the representative shift vector for that image pair. Through this displacement statistics mechanism, registration errors caused by insufficient local texture or mismatched points can be effectively suppressed, thereby enabling robust preliminary stitching of adjacent hologram pairs.

After obtaining the relative shifts for all adjacent image pairs, the system proceeds to global alignment and auto cropping. In this step, each hologram is mapped into a common global coordinate system according to the displacement results obtained from pairwise registration, thereby completing the overall stitching layout. However, due to practical shift errors, inconsistent image coverage, or local missing regions, the stitched hologram boundaries often contain black margins or invalid areas. Therefore, an auto cropping procedure is further applied to remove peripheral regions lacking valid information, ensuring that the most complete and continuous effective stitched area can be retained.

Finally, in the image blending stage, a linearly weighted fusion strategy [36] is adopted to reduce brightness discontinuities, seam artifacts, and local intensity jumps in the overlapping regions. Specifically, two accumulation matrices are constructed to store the summed pixel intensities and the summed weights, respectively. For each hologram to be stitched, its pixel values are first multiplied by the corresponding spatial weighting function and then accumulated into the intensity-sum matrix, while the same weights are accumulated into the weight-sum matrix. After all holograms have been added, the final blended image is obtained by dividing the summed intensities by the summed weights, which can be expressed as $P_{final}\left(x,y\right)=\sum_{i=1}^{n}P_{i}(x,y)W_{i}(x,y)/\sum_{i=1}^{n}W_{i}(x,y)$, where $P_{i}(x,y)$ denotes the pixel intensity of the *i*-th hologram at position (x,y), and $W_{i}(x,y)$ is the corresponding weighting coefficient. Through this weighted accumulation and normalization mechanism, discontinuities at stitching boundaries can be effectively reduced, thereby improving the visual consistency and structural integrity of the final extended FOV hologram.

In summary, the proposed multiple holograms stitching framework first establishes the topological relationships among holograms using the scanning coordinates, then estimates stable relative displacements through feature matching and pairwise registration within overlapping regions of adjacent holograms, and finally constructs the extended FOV hologram through global alignment, auto cropping, and image blending. The resulting stitched hologram serves as the basis for the subsequent holographic reconstruction and phase retrieval processes.

### 2.3. DKM-Based ROI Matching

**Figure 3:**
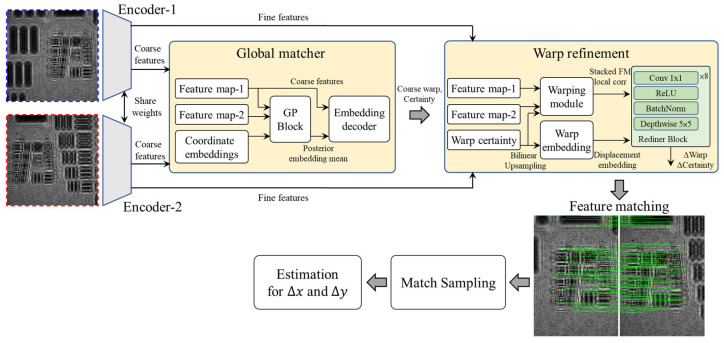
Operation flowchart of the DKM method. GP block: Gaussian process block, Conv: convolution.

In this study, the DKM method is employed for robust feature matching within overlapping regions of adjacent holograms, aiming to improve the stability and accuracy of correspondence estimation under challenging holographic conditions. The operation flowchart of DKM is shown in Figure 3, and consists of five main stages: shared-weight feature extraction, global matching, warp refinement, match sampling, and geometric estimation.

First, in the shared-weight feature extraction stage, the two input images to be matched are separately fed into two encoders with shared weights (Encoder-1 and Encoder-2) to extract multi-scale feature representations. This step generates both coarse features and fine features. The coarse features are used for subsequent global matching to establish the initial correspondences between the two images, whereas the fine features preserve more detailed local structural information for later matching refinement. Because the two images share the same feature extraction network, their feature representations are maintained within a consistent embedding space, thereby improving the reliability of the subsequent matching process.

Next, in the global matching stage, the initial global correspondences between the two images are established based on their coarse features. As shown in the figure, feature map-1, feature map-2, and coordinate embeddings are used as inputs, and are integrated and decoded through the Gaussian process block (GP block) and embedding decoder to estimate the correspondence relationship at the coarse feature level. The final outputs include an initial coarse warp and its corresponding certainty, which serve as the basis for subsequent local refinement. The purpose of this stage is to obtain the overall geometric correspondence and displacement trend between the two images in a lower-resolution feature space.

After the initial matching is completed, the model proceeds to the warp refinement stage to further improve pixel-level matching accuracy. This stage jointly uses the coarse warp and warp certainty obtained from the previous stage, together with the fine features extracted from the two input images. Specifically, the fine features and the initial warp information are separately fed into the warping module and the warp embedding module. The warp information is first upsampled to a higher resolution using bilinear upsampling and then transformed into the corresponding displacement embedding representation. The model then integrates the local correlation information with the displacement embedding results and feeds them into a refiner block composed of convolution, ReLU, BatchNorm, and depthwise convolution layers, thereby predicting the displacement correction term ΔWarp and the certainty correction term ΔCertainty. Through this refinement process, more accurate dense correspondences can be obtained from the initial coarse matching results.

After the refined dense matching results are obtained, the system enters the match sampling stage. In this step, the reliable correspondences are selected from all matching results according to the model outputs, including the dense correspondences and the certainty map. Because holographic images often exhibit low contrast, repetitive textures, and complex diffraction fringes, the matching results may still contain local mismatches or outliers. Therefore, the certainty map is used as a confidence criterion to retain only highly reliable matching samples for subsequent geometric parameter estimation. This step effectively reduces the influence of mismatched correspondences on the final registration result.

Finally, in the estimation stage, the filtered reliable matching points are fed into the estimation module to determine the final geometric relationship between the two images. In the hologram stitching application of this study, this geometric relationship mainly corresponds to the relative displacement of adjacent images in the x and y directions, i.e., (Δx,Δy), which serves as the basis for the subsequent pairwise registration, global alignment, and overall image stitching process.

Overall, DKM provides stable and dense correspondence information within the overlapping regions of adjacent holograms. When dealing with the common challenges in holographic images, such as low contrast, repetitive textures, and complex diffraction fringes, the coarse-to-fine matching framework, certainty-based match sampling, and subsequent displacement estimation process effectively improve the matching stability and registration accuracy of adjacent holograms. Detailed operational procedures and the complete model architecture of DKM can be found in ref. [35].

### 2.4. Multi-Depth Phase Retrieval

**Figure 4:**
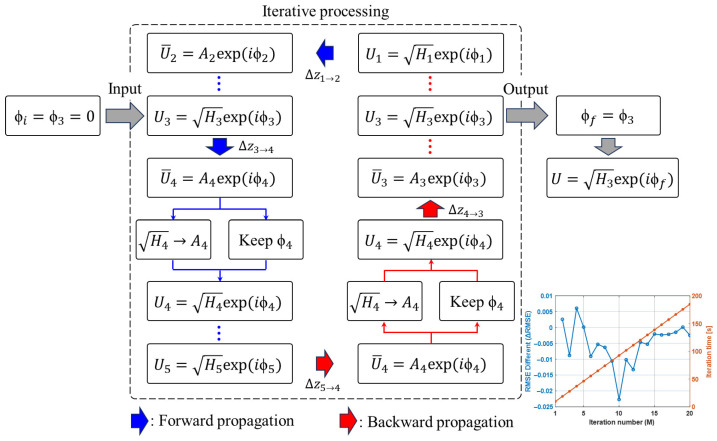
Flowchart of multi-depth phase retrieval.

The flowchart of the multi-depth phase retrieval method is shown in Figure 4. The algorithm takes as input a set of holograms recorded at multiple axial positions (z-planes), and a specific plane is selected as the initial reconstruction plane. In this study, the middle plane (the third plane) is chosen for initialization. The initial phase is set as $\phi_{i}=\phi_{3}=0$, and the initial complex wavefront at the third plane can therefore be expressed as ${\sqrt{H_{3}}exp(i\phi }_{3})$. The angular spectrum method is then used to propagate this field a distance ${∆z}_{3\to 4}$ to the fourth plane, resulting in a propagated field ${A_{4}exp(i\phi }_{4})$. Next, the amplitude of the propagated field is replaced with the measured hologram amplitude at the fourth plane, while retaining the phase information ${\sqrt{H_{4}}exp(i\phi }_{4})$. Following the same procedure, the wavefront is forward- and backward-propagated among all measurement planes, and the corresponding amplitude constraint is imposed at each plane. To evaluate the convergence behavior of the iterative reconstruction, the RMSE between the measured amplitude hologram at the $z_{3}$ plane and the amplitude image propagated back to the same plane after each iteration was calculated. All amplitude images were normalized before RMSE evaluation. In addition, the RMSE difference between two consecutive iterations was analyzed to assess the change in reconstruction error during the iterative process. The RMSE difference between two consecutive iterations was further defined as ${\Delta RMSE}_{M}\;=\;({RMSE}_{M}\;-\;{RMSE}_{M-1})$, where M denotes the iteration number. As shown in Figure 4, the RMSE difference decreases rapidly during the early iterations and becomes close to zero after approximately 10 iterations, indicating that the reconstruction process has reached a stable convergence state. Since the computational time increases approximately linearly with the number of iterations, further increasing the iteration number provides only limited improvement in reconstruction error but increases the computational cost. Therefore, 10 iterations were selected in this study to balance reconstruction stability and computational efficiency. After the iterative update process converges, the reconstructed phase at the third plane is obtained as $\phi_{3}=\phi_{f}$. By combining this phase with the measured hologram amplitude $\sqrt{H_{3}}$ at the same plane, the final complex wavefront can be expressed as ${\sqrt{H_{3}}exp(i\phi }_{f})$. By integrating the complementary information provided by multiple depth planes, the multi-depth phase retrieval method improves the stability and accuracy of phase reconstruction, while mitigating the twin-image artifacts and noise that commonly affect single plane reconstruction. After iterative updates over all depth planes, a more accurate complex wavefront can be recovered at the target reconstruction plane, thereby enabling the reconstruction of both the amplitude and quantitative phase images of the sample.

## 3. Experimental Setup and Results

**Figure 5:**
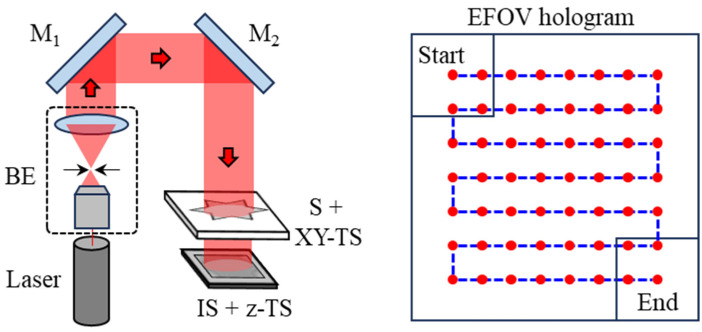
Experimental setup of lensless digital holography. In EFOV hologram, the red dots indicate the center positions of the recorded holograms, while the blue dashed line represents the serpentine scanning path. BE: beam expander, M: mirror, S: sample, TS: translation stage, IS: image sensor.

The experimental configuration of the lensless digital holography system used in this study is shown in Figure 5. A He–Ne laser (Uniphase, St. Charles, IL, USA; model: 1145P; λ = 632.8 nm; 21 mW) served as the illumination light source. The laser beam was first expanded and collimated by a beam expander (BE). The expanded collimated beam was then incident on the sample, and the resulting defocused holographic diffraction images were recorded by an image sensor (Pixoel, Taipei City, Taiwan; model: U3-34L0XCP-M-GL; pixel number: 3552 × 3552; pixel size: 2 μm). The distance between the sample and the image sensor, denoted as $z_{1}$, was set to 114 mm. In order to record multi-depth defocused holograms for subsequent multi-depth phase retrieval, the image sensor was translated along the optical axis using a motorized *z*-axis translation stage (Sigma Koki, Tokyo, Japan; model: SGSP20-20). The axial spacing between adjacent depth planes is set to 1 mm, and holograms were recorded at a total of five different axial planes. This parameter selection is based on the requirement of sufficient propagation diversity for multi-depth phase retrieval. When the axial spacing is too small, the variation in diffraction patterns between adjacent planes becomes limited, which weakens the reconstruction constraints and may reduce the stability of the phase retrieval process. Therefore, the axial spacing should exceed the effective depth of field of the system to ensure adequate intensity variation across different planes. Based on the depth of field estimation $\lambda /{NA}^{2}$, where the effective numerical aperture (NA) of a single hologram is approximately 0.0306, the depth of field is calculated to be about 676 μm. Accordingly, an axial spacing of 1 mm was adopted in this study to ensure sufficiently distinct diffraction patterns between adjacent planes, while also maintaining experimental convenience and system feasibility. The number of measurement planes was determined as a trade-off between reconstruction robustness and computational cost. Experimental observations indicate that five planes provide sufficient constraints for stable convergence of the phase retrieval process. Although increasing the number of planes may introduce additional measurement information, it also leads to higher acquisition and computational burdens. Among them, the third plane, $z_{3}$ = 116 mm, was selected as the reconstruction plane for the subsequent phase retrieval process. To acquire lateral sample information, a high-precision motorized x-y translation stage (Thorlabs, Newton, NJ, USA; model: MLS203-1) was used to scan the sample. According to the manufacturer’s specifications, the translation stage provides a bidirectional repeatability of 0.25 μm and an absolute on-axis accuracy of <3 μm. In this work, the center-to-center displacement between adjacent FOVs was set to 3 mm, corresponding to an FOV overlap ratio of 57.7%. To achieve efficient extended FOV hologram acquisition, the sample was scanned in a row-by-row serpentine pattern, starting from the upper-left position and ending at the lower-right position. In Figure 5, the red dots indicate the hologram centers corresponding to each scanning position, whereas the blue dashed line represents the serpentine scanning path of the sample. This scanning strategy reduces unnecessary stage return motion between adjacent rows and thereby improves the acquisition efficiency.

**Figure 6:**
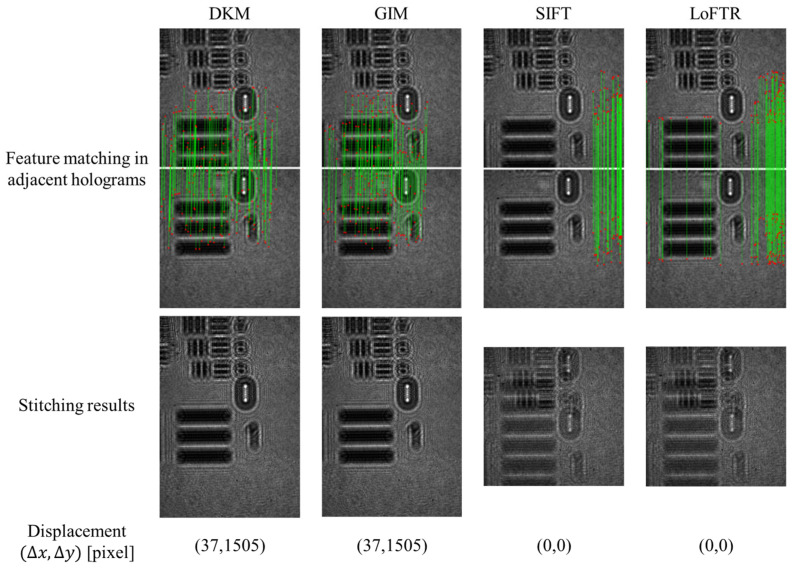
Comparison of feature matching results for hologram stitching using DKM. GIM, SIFT, and LoFTR. The green lines represent the matched feature correspondences between paired points in adjacent holograms, and the red points denote the detected feature locations.

To evaluate the suitability of feature matching methods for hologram stitching, Dense Kernelized Matching (DKM) was compared with Generalizable Image Matcher (GIM) [37], Scale-Invariant Feature Transform (SIFT) [38], and Detector-Free Local Feature Matching with Transformers (LoFTR) [39]. The comparison focuses on matching reliability, displacement estimation accuracy, robustness under low-contrast conditions, and computational efficiency. Figure 6 presents the comparison of feature matching, displacement estimation, and stitching performance for these methods. The first row shows the feature matching results, where (Δx, Δy) denotes the estimated relative translation, and the second row shows the corresponding stitching results. It is noted that accurate displacement estimation depends not only on the number of matches but also on their spatial consistency and the dispersion of displacement vectors. As shown in Figure 6, DKM and GIM estimate a displacement of (37, 1505), which is consistent with the physical shift between adjacent acquisitions. In particular, DKM produces dense and spatially well-distributed correspondences with low dispersion, leading to stable displacement estimation. In contrast, SIFT and LoFTR yield inaccurate displacement estimation (0, 0), which can be attributed to insufficient or spatially inconsistent matches. This behavior is closely related to the characteristics of holographic images, which typically exhibit low contrast and highly repetitive fringe patterns, reducing feature discriminability. Under such conditions, SIFT tends to produce sparse and unevenly distributed matches, while LoFTR may generate ambiguous correspondences with higher variability. These factors lead to less reliable displacement estimation. The impact of matching performance is further reflected in the stitching results. DKM and GIM achieve accurate alignment without visible artifacts, whereas SIFT and LoFTR show noticeable misalignment and ghosting effects due to incorrect displacement estimation. In terms of computational performance, DKM requires 15.02 GB of memory and 163.38 s of computation time, compared to 31.92 GB and 248.95 s for GIM, and 42.14 GB and 566.17 s for LoFTR. Although SIFT is more computationally efficient (3.92 GB and 146.91 s), its matching reliability is insufficient for holographic fringe images. Overall, DKM provides a favorable trade-off between robustness and computational cost, and is therefore adopted in the proposed framework.

**Figure 7:**
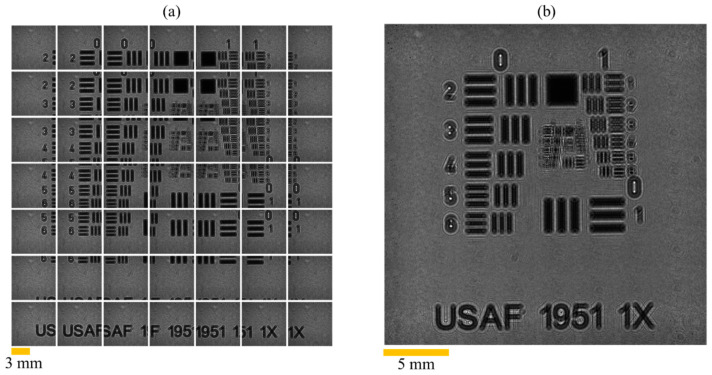
Multiple recorded holograms and EFOV image. (**a**) Multiple holograms acquisition. (**b**) EFOV hologram.

The multiple holograms acquisition and the corresponding extended FOV (EFOV) stitching results are shown in Figure 7. In this experiment, a USAF 1951 resolution target was used as the test sample to evaluate the effectiveness of the proposed method in EFOV stitching and resolution enhancement. Because the USAF 1951 target contains well-defined and regularly distributed line-pair structures, it is widely used for validating the resolution and imaging quality of optical imaging systems. It is therefore suitable for assessing the detail preservation capability and overall consistency of the stitched image across different regions. In this study, a total of 7 × 7 holograms were recorded. The FOV of a single hologram was 7.1 × 7.1 mm^2^ with the matrix size of 3552 × 3552 pixels. After multiple holograms stitching, an extended image with a final FOV of 24.6 × 24.6 mm^2^ and the matrix size of 12,300 × 12,300 pixels was obtained. Compared with a single hologram, the total imaging area after stitching was increased by approximately 12-fold, demonstrating that the proposed method can effectively expand the observable range of the system.

**Figure 8:**
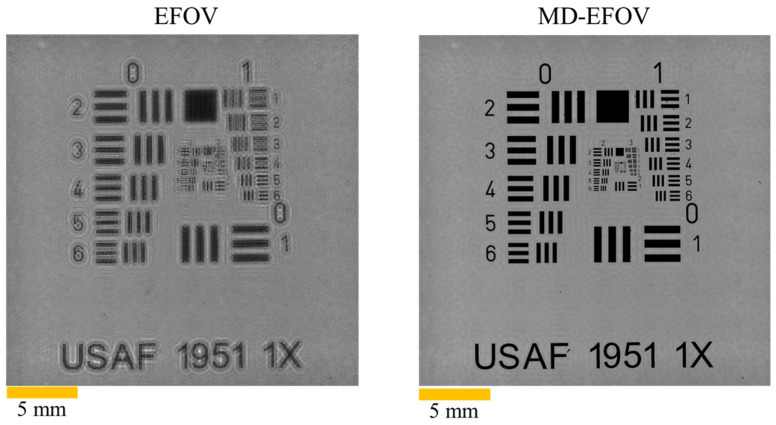
Reconstructed results of EFOV and MD-EFOV.

After the holograms at the five different axial planes were stitched, the multi-depth phase retrieval method was applied to reconstruct the sample wavefront, thereby recovering the phase information of the sample while simultaneously suppressing the twin-image artifacts commonly observed in conventional in-line holographic reconstruction, leading to improved overall reconstruction quality. In this study, the $z_{3}$ plane (*z* = 116 mm) was selected as the reconstruction plane. Figure 8 presents the reconstructed results of EFOV reconstruction and MD-EFOV reconstruction. Here, the EFOV reconstruction refers to the reconstruction result obtained without multi-depth phase retrieval, whereas the MD-EFOV reconstruction denotes the result after applying the multi-depth phase retrieval process. The results clearly show that the MD-EFOV image exhibits better contrast and image clarity than the EFOV reconstruction and that the background diffraction interference caused by twin-image artifacts is effectively suppressed after multi-depth phase retrieval. In this phase retrieval stage, a total of 10 iterations are performed.

**Figure 9:**
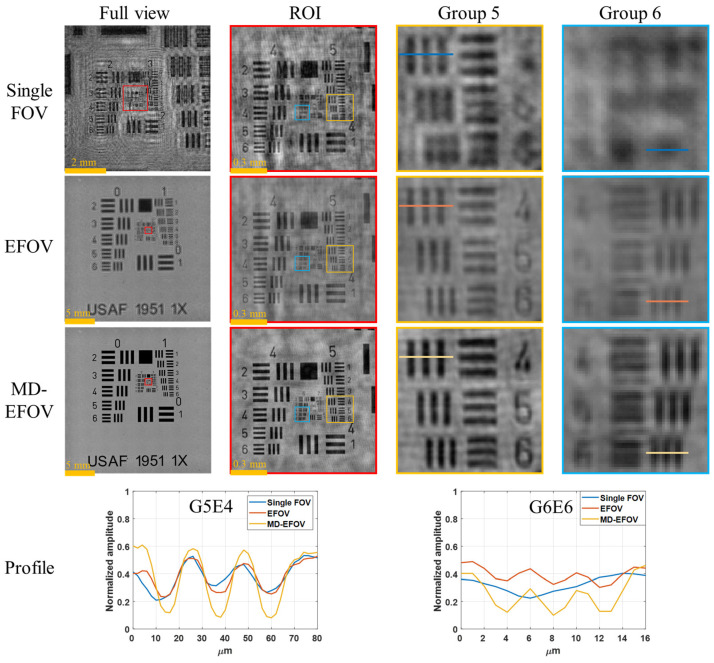
Comparison results of single FOV, EFOV and MD-EFOV using resolution target. ROI: region of interest.

In terms of effective reconstruction resolution, EFOV holographic imaging has the potential to improve the effective resolving capability compared with a single FOV image, because it integrates complementary diffraction information captured from different FOVs. Figure 9 shows the resolution performance of the single FOV reconstruction, EFOV reconstruction, and MD-EFOV reconstruction. The results show that the single FOV image can only resolve up to Group 5 Element 4 (G5E4, 11.05 μm), representing the baseline resolution performance of the lensless digital holography system. At G5E4, the contrasts of the single FOV, EFOV, and MD-EFOV reconstructions are 0.21, 0.25, and 0.48, respectively. In contrast, both the EFOV reconstruction and MD-EFOV reconstruction can further resolve Group 6 Element 6 (G6E6, 4.38 μm), with corresponding contrasts of 0.10 and 0.17, respectively, whereas the single FOV reconstruction fails to resolve these finer structures. The quantitative evaluation is based on line profile analysis, in which the intensity modulation between adjacent bright and dark fringes is characterized using contrast. Because the USAF 1951 resolution target consists of periodic line-pair structures, the measured fringe contrast can be used to approximate the system modulation at the corresponding spatial frequency. The resolution criterion adopted in this study is based on the commonly used MTF10 standard [40,41] in optical imaging, where a modulation level of approximately 10% is considered to be close to the resolution limit. Since the USAF resolution target provides high-contrast line-pair features and the reconstructed images were normalized prior to evaluation, the measured fringe contrast can be regarded as an approximation of the system modulation at the corresponding spatial frequency. Accordingly, a contrast of approximately 0.10 indicates that the system is near its resolution limit, while higher contrast values correspond to more reliable feature discrimination. Based on this criterion, the EFOV result at G6E6 is close to the resolution limit, whereas the MD-EFOV reconstruction exhibits a higher contrast, indicating improved discrimination of high spatial frequency structures. These results indicate that multiple holograms stitching improves the effective reconstruction resolution of the system by integrating complementary diffraction information acquired at different lateral positions. Moreover, the MD-EFOV reconstruction exhibits better image quality than the EFOV reconstruction. Although the EFOV reconstruction already improves the effective resolution, it is still susceptible to twin-image interference, which degrades local detail visibility and overall contrast performance. By comparison, the MD-EFOV reconstruction effectively suppresses background diffraction interference and further enhances image contrast and structural clarity. It should be noted that, in lensless digital holography, the achievable resolution is not solely determined by the sensor sampling condition. While the sensor pixel size defines the Nyquist sampling limit, the effective system resolution is jointly constrained by multiple factors, including the illumination wavelength (*λ*), propagation distance (*z*), FOV (*L*), hologram quality, and reconstruction accuracy. According to ref. [18], the theoretical spatial resolution of lensless digital holography can be expressed as $\lambda \sqrt{{4z}^{2}+L^{2}}/2L$. Based on the system parameters used in this study, the theoretical resolution of EFOV and MD-EFOV are estimated to be approximately 3 μm, whereas the experimentally achieved resolution is 4.38 μm. The discrepancy between theoretical and experimental results can be attributed to non-ideal factors in practical measurements, such as noise, limited hologram quality, and reconstruction errors. Overall, these results demonstrate that the proposed method not only extends the imaging FOV of lensless digital holography but also simultaneously improves the effective reconstruction resolution and overall image quality.

To further clarify the mechanism behind the observed improvement, it is important to distinguish it from conventional resolution enhancement techniques. In lensless digital holography, holograms acquired at different lateral positions correspond to shifted sampling of the object wavefront, resulting in variations in the captured diffraction patterns. These measurements contain complementary diffraction information. When multiple FOVs are combined through stitching and numerical reconstruction, this complementary information is integrated, leading to improved visibility and discrimination of high-spatial-frequency features. From a spatial-frequency perspective, this process enhances the completeness of the captured frequency information, rather than extending the system bandwidth. Therefore, the observed improvement should be interpreted as an enhancement in effective reconstruction resolution, rather than an intrinsic increase in optical resolution. It should also be emphasized that the proposed method does not rely on subpixel shifts or synthetic aperture configurations, and thus differs from conventional super-resolution or synthetic aperture imaging approaches.

In addition to the reconstruction performance, the computational cost of the proposed MD-EFOV framework was evaluated. All computations were performed on an NVIDIA GB10 GPU with 128 GB memory and a memory bandwidth of 273.2 GB/s. For a single z-plane, hologram stitching with an input size of 3552 × 3552 × 49 required approximately 15.02 GB of memory and 163.38 s of computation time. The multi-depth phase retrieval process, with an EFOV size of 12,300 × 12,300 pixels over five depth planes, required approximately 30.43 GB of memory and 92.5 s for 10 iterations. The computational cost mainly arises from large-scale hologram stitching and repeated wavefront propagation in the iterative phase retrieval process. Although the current implementation is not intended for real-time processing, the measured computation time indicates that the proposed framework is feasible for off-line large area holographic reconstruction. It should be noted that the current implementation has not been fully optimized for computational efficiency, and the reported performance reflects a straightforward implementation of the proposed framework. Further improvements, including parallelization strategies and memory optimization, will be explored in future work to enhance practical applicability.

**Figure 10:**
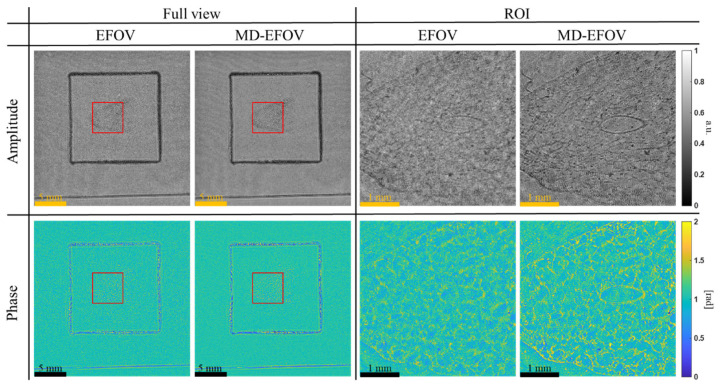
Comparison results of EFOV and MD-EFOV using injected lung vascular specimen. Red box represents the locally magnified ROI.

Figure 10 compares the amplitude and phase reconstructions of a commercially available injected lung vascular specimen (Telescopes, Taipei City, Taiwan; B-100P) using EFOV and MD-EFOV methods. In the full view images, both methods successfully reconstruct the overall morphology and the main structural distribution of the specimen, while the extended FOV image also reveals the spatial relationship of the sample within the entire FOV. The black box indicates the coverage area of the cover glass, and the sample is located in its central region, demonstrating that EFOV reconstruction is useful for providing more complete large area sample information. By contrast, in the locally magnified ROI (red box), the MD-EFOV method exhibits improved local contrast and structural discrimination compared with the EFOV result. In the amplitude image, the tissue texture and the contours of oval-like local structures can be visualized more clearly, with enhanced edge contrast and reduced background fluctuations. In the phase image, the corresponding boundaries and fine structural variations are also more distinct than those in the EFOV reconstruction. These improvements can be attributed to the incorporation of multi-depth phase retrieval, which provides additional reconstruction constraints that help suppress twin-image interference and background diffraction artifacts. As a result, the MD-EFOV reconstruction enhances the visibility of fine microstructures in large area biological specimens, demonstrating improved reconstruction quality under practical imaging conditions.

To clarify the novelty, the key contributions of this study are summarized as follows:

(1) A unified MD-EFOV framework that jointly integrates multi-FOV stitching and multi-depth phase retrieval at the hologram level, enabling simultaneous improvement in FOV and reconstruction fidelity.
(2) A diffraction-domain information integration mechanism, which differs from synthetic aperture and pixel super resolution, as it does not rely on subpixel shifts, angular diversity, or bandwidth expansion.
(3) A robust stitching strategy specifically designed for defocused holographic fringes using DKM, addressing a scenario where conventional feature-based methods fail.
(4) Demonstration of large area holographic imaging with enhanced effective reconstruction capability. The proposed framework enables extended FOV imaging while improving local contrast, structural visibility, and effective resolving capability through the integration of complementary diffraction information.

Overall, the experimental results demonstrate that the proposed MD-EFOV method can simultaneously achieve large area imaging and enhanced local structural visualization. Multiple holograms stitching (Figure 7) effectively extends the observable area, preserving the overall morphology and spatial distribution of the sample, while multi-depth phase retrieval (Figure 8) enhances local contrast and structural discernibility without compromising the large FOV advantage. Resolution target analysis (Figure 9) and biological sample reconstructions (Figure 10) further validate that MD-EFOV not only extends the imaging range but also improves the visibility of fine structures in complex specimens. These results confirm that the proposed method can reliably capture both global context and microstructural detail, highlighting its strong potential for practical large area imaging and high-resolution biological imaging applications.

## 4. Conclusions

In this study, a large FOV lensless digital holography imaging method combining multiple holograms stitching and multi-depth phase retrieval was proposed to address the limited imaging area and twin-image interference in conventional lensless digital holography. Through x-y scanning and hologram stitching, the FOV was extended from 7.1 × 7.1 mm^2^ to 24.6 × 24.6 mm^2^, corresponding to an approximately 12× increase in imaging area. By integrating complementary diffraction information from different FOVs, the proposed method further improved the effective reconstruction resolution. Using a USAF 1951 resolution target, the single FOV reconstruction could resolve only up to G5E4 (11.05 μm), whereas both the EFOV and MD-EFOV reconstructions could further resolve G6E6 (4.38 μm). This result demonstrates that multi-FOV hologram stitching enhances the effective resolving capability of the reconstruction, rather than simply enlarging the imaging area. Furthermore, multi-depth phase retrieval effectively suppressed twin-image interference and improved image contrast and detail visibility, with the MD-EFOV reconstruction showing better contrast than the EFOV reconstruction. Further validation using a biological section specimen demonstrated that the proposed MD-EFOV method not only enables large area observation of the overall sample morphology but also enhances the visibility of local tissue structures and phase information. Overall, the proposed method achieves large FOV, high-resolution, and high-quality holographic imaging while preserving the simple optical architecture of lensless digital holography, showing strong potential for large area microstructure inspection, biomedical imaging, and complex specimen imaging. Future work will focus on improving computational efficiency and scalability through algorithm optimization, GPU acceleration, block-wise processing, and memory management. In addition, the influence of phase retrieval parameters, such as the number of axial planes, axial spacing, iteration number, and noise conditions, will be systematically investigated to further enhance the robustness, reconstruction quality, and practical applicability of the proposed framework.

## Data Availability

The dataset underlying the results presented in this paper is not publicly available at this time but may be obtained from the authors upon reasonable request.
